# Factors associated with major structural birth defects among newborns delivered at Muhimbili National Hospital and Municipal Hospitals in Dar Es Salaam, Tanzania 2011 – 2012

**DOI:** 10.11604/pamj.2015.20.153.4492

**Published:** 2015-02-18

**Authors:** Rogath Saika Kishimba, Rose Mpembeni, Janneth Mghamba

**Affiliations:** 1 Field Epidemiology and Laboratory Training Programme (FELTP), Tanzania; 2 Muhimbili University of Health and Allied Sciences (MUHAS), Tanzania; 3 Ministry of Health and Social Welfare, Tanzania

**Keywords:** Birth defects, risk factors, newborns, mothers, mortality, Tanzania

## Abstract

**Introduction:**

Ninety-four percent of all birth defects and 95% of deaths due to the birth defects occur in low and middle income countries, Tanzania among them. In Tanzania there are currently limited birth defects prevention strategies in place due to limited information on factors associated with the occurrence of birth defects.

**Methods:**

We conducted a case control study that included newborns born from October, 2011 through February, 2012 at 4 participating hospitals. A case was defined as any newborn of a Dar es salaam resident with a neural tube defect, orofacial clefts, limb reduction defects or musculo-skeletal defects (SBD) born during the study period. A control was defined as the next three newborns (delivered after the case) without birth defects. Univariate, bivariate and multivariate analysis were done using Epi Info version 3.5.1.

**Results:**

A total of 400 newborns participated in the study, 100 cases and 300 controls. Factors associated with higher odds of a SBD included maternal fever (adjusted odds ratio (AOR) = 1.99; 95% confidence interval (CI): 1.14-3.52), maternal hypertension (AOR = 3.99; 95% CI: 1.67-9.54), and low birth weight (AOR = 3.48; 95% CI: 1.77-6.85). Antimalarial use during pregnancy was protective (AOR = 0.48; 95% CI: 0.28-0.84). Folic acid supplementation was protective only in bivariate analysis (OR = 0.56; 95% CI: 0.32-0.96).

**Conclusion:**

Maternal fever, hypertension, and low birth weight are associated with higher odds of SBD. Antimalarial use during pregnancy was associated with lower odds of SBD. Early screening of pregnant mothers for hypertension and other causes of low birth weight may reduce SBD in Dar Es Salaam.

## Introduction

A birth defect is an abnormality of structure or function which originates during intrauterine development and is evident before birth, at birth or manifests later in life. Structural birth defects (SBD) may be clinically obvious at birth [1]. The most common major structural birth defects include congenital heart disease (CHD), neural tube defects, orofacial clefts and limb reduction defects [1, 2]. Birth defects have adverse effects on the wellbeing and survival of children born with those anomalies. Almost all birth defects (94%) and deaths due to the birth defects among children (95%) occur in low and middle income countries [2]. The March of Dimes (MOD) and World Health Organization (WHO) estimate that at least 3.3 million children under age of five years die from serious genetic or partly genetic birth defects and 3.2 million of those who survive may be disabled for life without appropriate care; exacting a severe human and economic toll on those affected, their families and their communities [2, 3]. In Tanzania, it is estimated that the prevalence of birth defects is 60.5 per 1,000 live births [3]. Studies done at Muhimbili National Hospital neonatal unit in Dar es Salaam have shown a birth defects prevalence of 3.3%, with eight percent of the overall mortality in the neonatal unit attributed to birth defects [4, 5]. However, there is currently no surveillance system in Tanzania to describe the population prevalence of birth defects.

Birth defects due to teratogens are among the most preventable defects and are more likely to be found in middle and low income countries compared to high income countries [6, 7]. This is because pregnancies in these countries are more likely to be exposed to potential teratogens and other risk factors [6, 7]. Potential teratogens/factors that have been identified in developing countries as contributors to birth defects include low socioeconomic and educational levels, malnutrition (mineral and vitamin deficiencies), intrauterine infections, lack of environmental protection policies, environmental pollution, unsafe working conditions during pregnancy, access to medicines without medical indication or prescription (self-medication), and common use of home remedies of unknown composition [7]. Clearly documenting the magnitude of known risk factors that influence the occurrence of birth defects will help inform developing preventive strategies [6, 7]. In developed countries, there are well established prevention programs which address non genetic factors influencing birth defects. However, Tanzania's ability to establish prevention strategies for birth defects is hampered by limited information on the magnitude and factors that influence the occurrence of birth defects. This study helps increase understanding of the magnitude of known risk factors, hence contributing to developing data driven birth defects prevention activities and demonstrating the value of developing a surveillance system for birth defects in Tanzania.

## Methods

This study, a retrospective case-control study nested within that birth cohort, included all newborns delivered October, 2011 through February, 2012 in Dar es Salaam at Muhimbili National Hospital (MNH) and the three Municipal hospitals (Temeke, Mwananyamala and Amana). None of these hospitals are referral hospitals for mothers identified with a birth defects affected fetus. Sample size was calculated using the Fleiss formula with a case control ratio of 1:3; assuming the proportion of controls exposed was 54% [8], the minimum odds ratio to be detected was 2.0, power of 80% and significance level of 95%. Computed sample size was 412, (Cases 103 and Controls 309). A case was defined as any newborn of a Dar es Salaam resident with a neural tube defect, orofacial clefts, limb reduction defects or musculo-skeletal defects born at participating hospitals during the study period. A control was defined as the next three newborns (delivered after the case) to Dar es Salaam residents without birth defects born at participating hospitals during the study period. Newborns who met the case/control definition and whose mother's gave informed consent were included in the study. Newborns that met the case/control definition, but whose mothers could not be interviewed because they were very sick, emotionally upset, mute or died post-delivery were excluded from the study. A Dar es Salaam resident mother was considered to be any pregnant woman who lived in Dar es Salaam for the 6 months prior to delivery.

To avoid duplication of interviews when a mother delivered more than one newborn, the following rules were implemented: if all newborns met the case definition, only the first born was included in the study; if only one newborn met the case definition while the other did not, only the case was included in the study; if all newborns met the control definition, only the first born was included in the study. A structured questionnaire was used to interview mothers whose newborns participated in the study. The information included maternal and paternal demographic data and also information on factors that influence structural birth defects. The outcome (major structural birth defects, MSBD) was defined as any newborn with diagnosis of a neural tube defect, an orofacial cleft, a musculo-skeletal defect, or any combination of these defects. Bivariate and multivariate analyses were employed, and factors which had a p-value of ≤ 0.2 in the bivariate analysis were included in the multivariate analysis. P-values of less than or equal to 0.05 were considered statistically significant. Newborns with ambiguous genitalia were excluded from analysis. Data were analysed using Epi Info version 3.5.1.

## Results

A total of 400 newborns participated in the study - 100 cases and 300 controls - with a 97.1% consented to be interviewed.

### Socio demographic characteristics of the study sample

Out of the 400 mothers whose newborns were included, 310 (77.5%) were ages 20 to 35 years with a mean age of 25.9±5.9 years and 361(90.3%) were married. More than half (73.3%) of the 400 mothers had primary education (Table 1). None of the socio demographic characteristics were statistically significantly different between cases and controls.

**Table 1:** Socio demographic characteristic of case and control groups

Characteristics	Cases (N = 100)	Controls (N = 300)	Total (N = 400)	p-value
**Study Site**				
Muhimbili National Hospital	14 (14.0%)	42 (14.0%)	56 (14.0%)	
Temeke Hospital	35 (35.0%)	105 (35.0%)	140 (35.0%)	
Mwananyamala Hospital	34 (34.0%)	102 (34.0%)	136 (34.0%)	
Amana Hospital	17 (17.0%)	51 (17.0%)	68 (17.0%)	0.99
**Maternal age(years)**				
<20	15 (15.0%)	40 (13.3%)	55 (13.7%)	
20 – 35	75 (75.0%)	235 (78.3%)	310 (77.5%)	
>35	10 (10.0%)	25 (8.4%)	35 (8.8%)	0.78
**Paternal age (years)**				
< 25	13 (13.0%)	26 (8.7%)	39 (9.8%)	
25-30	40 (40.0%)	124 (41.3%)	164 (41.0%)	
>30	47 (47.0%)	150 (50.0%)	197(49.2%)	0.45
**Maternal education**				
None	7 (7.0%)	25 (8.3%)	32 (8.0%)	
Primary school	74 (74.0%)	219 (73.0%)	293 (73.3%)	
Secondary school or higher	19 (19.0%)	56 (18.7%)	75 (18.7%)	0.91
**Maternal marital status**				
Non married	11 (11.0%)	28 (9.3%)	39 (9.7%)	
Married	89 (89.0%)	272 (90.7%)	361(90.3%)	0.63
Newborn sex*				
Female	48 (49.5%)	166 (55.3%)	214 (53.5%)	
Male	49 (50.5%)	134 (44.7%)	183 (45.8%)	0.32

* Three newborns had ambiguous sex and so were excluded

### Factors associated with major structural birth defects

Bivariate and multivariate analyses were employed to assess associations between factors and MSBD (Table 2). Factors significantly associated with MSBD included maternal fever (adjusted odds ratio (AOR) = 1.99; 95% confidence interval (CI): 1.14-3.52), maternal hypertension (AOR = 3.99; 95% CI: 1.67-9.54), and low birth weight (AOR = 3.48; 95% CI: 1.77-6.85). Antimalarial use during pregnancy had a protective association (AOR = 0.48; 95% CI: 0.28-0.84), although the gestation at which the mother took antimalarial was not ascertained during interview. Folic acid supplementation during pregnancy irrespective of gestational age had a protective association, but only in bivariate analysis (crude odds ratio (COR) = 0.56; 95% CI: 0.32-0.96). Paternal age, consanguineous marriage, maternal HIV/AIDS, maternal Syphilis, maternal use of: alcohol, multivitamin, antiepileptic, antihypertensive, antibiotics and antiretroviral drugs during pregnancy, newborn sex and prematurity were not significantly associated with major structural birth defects.

**Table 2:** Crude and adjusted odds ratios for factors associated with birth defects

Characteristics	Case, N = 100 n (%)	Control, N = 300 n (%)	COR(95% CI)	AOR(95% CI)
**Maternal age(years)**				
<20	15 (15.0)	40 (13.3)	Ref	
20 – 35	75 (75.0)	235 (78.3)	0.85 (0.45-1.63)	0.95 (0.42-2.13)
>35	10 (10.0)	25 (8.4)	1.07 (0.42-2.73)	1.19 (0.37-3.87)
**Unexplained maternal fever**				
No	67 (67.0)	242 (80.7)	Ref	
Yes	33 (33.0)	58 (19.3)	2.17 (1.30-3.61)	1.99 (1.14-3.52)*
**Malaria**				
No	60 (60.0)	211 (70.3)	Ref	
Yes	40 (40.0)	89 (29.7)	1.58 (0.99-2.53)	1.02 (0.55-1.92)
**Epilepsy**				
No	98 (98.0)	299 (99.7)	Ref	
Yes	2 (2.0)	1 (0.3)	6.1 (0.55-68.02)	7.60 (0.68-85.41)
**Hypertension**				
No	86 (86.0)	289 (96.3)	Ref	
Yes	14 (14.0)	11 (3.7)	4.28 (1.87-9.77)	3.99 (1.67-9.54)*
**Folic acid intake**				
No	25 (25.0)	48 (16.0)	Ref	
Yes	73 (73.0)	252 (84.0)	0.56 (0.32-0.96)	0.59 (0.33-1.06)
**Antimalaria**				
No	31 (31.0)	61 (20.3)	Ref	
Yes	69 (69.0)	239 (79.7)	0.57 (0.34-0.94)	0.48 (0.28-0.84)*
**Newborn birth weight (grams)**				
≥2500	77 (77.0)	279 (93.0)	Ref	
<2500	23 (23.0)	21 (7.0)	3.97 (2.09-7.55)	3.48 (1.77-6.85)*
**Newborn sex**				
Female	47 (47.0)	166 (55.3)	Ref	
Male	53 (53.0)	134 (44.7)	1.40 (0.89-2.20)	1.54 (0.94-2.49)
**Prematurity**				
(Born <37 weeks)				
No	55 (55.0)	189 (63.0)	Ref	
Yes	45 (45.0)	111 (37.0)	1.39 (0.88-2.20)	1.55 (0.94-2.57)
**Paternal age(years)**				
25-30	40 (40.0)	124 (41.3)	Ref	
<25	13 (13.0)	26 (8.7)	1.55 (0.73-3.29)	1.39 (0.57-3.45)
>30	47 (47.0)	150 (50.0)	0.97 (0.59-1.58)	0.94 (0.54-1.64)

* Statistically significant at p ≤ 0.05

## Discussion

Maternal hypertension, maternal fever during pregnancy, and low birth weight newborns had higher odds of developing structural birth defects, after accounting for other factors; while antimalarial use during pregnancy was associated with lower odds of structural birth defects. Mothers who reported fever during pregnancy had significantly higher odds s of giving birth to babies affected with congenital anomalies even after adjusting for other factors. Studies conducted in a variety of settings around the world have observed a significant association between maternal fever and birth defects that is consistent with our findings [9–14]. A population based case control study done in Shanxi province Northern China on risk factors for neural tube defects found that a history of fever during the periconceptional period was associated with almost a threefold increase in risk for neural tube defects, which persisted even after controlling for other covariates [15, 16]. We also found that hypertensive pregnant mothers had significantly higher odds of giving birth to children affected with birth defects than the non-hypertensive mothers. Our results are consistent with findings of a study done in the USA looking at maternal factors and disparities associated with oral clefts [17]. This may be explained by the fact that hypertension in pregnancy compromises uteroplacental perfusion, interfering with foetal development. Hypertension in pregnancy had also been shown to significantly increase the risk for other birth defects such as hypospadias, aesophageal atresia/stenosis and congenital heart disease [18–21]. In this study, use of an antimalarial during pregnancy had a significant protective association with birth defects that remained after controlling for other covariates. While in this study antimalarial use was verbally reported and timing as well as duration was not ascertained, there is biological plausibility. Malaria parasites require folic acid for replication, potentially competing with the fetus which also requires folic acid to support embryonic growth. A study done to evaluate toxicological consequences of chloroquine and ethanol on the developing rat fetus has shown a teratogenic effect of antimalarials resulting in structural birth defects such as cleft palate, wrist drop, clubbed foot and brain liquification [22]. However there is currently insufficient evidence on the potential teratogenic effects of antimalarials other than tetracycline at standard doses [23]. Mothers who took folic acid supplementation tablets during pregnancy were more than 50% less likely to have children with MSBD as compared to those who did not take supplemental folic acid tablets. Though, the association with folic acid supplementation was not significant in our multivariate analysis, the direction and magnitude of association is consistent with other studies that have shown folic acid supplementation is protective against birth defects [24–30]. The lack of a statistically significant finding in our study might be due to the omission of about one third of women who took multivitamins (which might also contain folic acid), as this is not in Tanzania's standard Reproductive and Child Health Antenantal package recommendation. In places where preconception or periconception use of folic acid supplements is absent, high rates of neural tube defects have been described [24]. In contrast, places where preconception use of folic acid supplementation has been implemented have shown decreased rates of neural tube defects [26, 28]. In our environment, preconception use of folic acid supplementation is an effective approach as 68.9% of pregnancies are wanted [31]. Periconceptional folic acid supplementation is in practice in Tanzania for all pregnant women attending antenatal clinics to counteract anaemia in pregnancy; however, because the neural tube closes in the first 28 days of conception, a time when most women do not yet recognize that they are pregnant, periconceptional folic acid likely only provides limited protection against neural tube defects in Tanzania.

There was an association between newborn birth weight and MSBD. Low birth weight babies (<2500 grams) had higher odds of congenital defects compared to normal birth weight babies (≥2500 grams). This can be potentially explained by several factors. Some birth defects included in our study may increase the risk of polyhydramnios which can result in preterm labour [32–34]. Preterm labour and birth defects may also share other risk factors such as maternal fever, maternal hypertension and maternal infections. In contrast to our findings, preterm infants are at increased risk of birth defects [32, 35]. A significant association which we found between low birth weight and birth defects can not necessarily be implied to a relationship between prematurity and birth defects due to the fact a third variable i.e. intrauterine growth retardation can distort this relationship [35]. In contrast to our findings, other studies conducted in a variety of countries have found an association between structural birth defects and advanced maternal age (≥35 years), paternal age >30 years, consanguineous marriage, alcohol consumption and antiepileptic drugs use during pregnancy [36–39]. This might be due to differences in the study populations in terms of included behaviors and diseases.

### Strengths and limitations of the study

In Dar es sallam, 90.2% of pregnant mothers deliver at a health facility and majority (72%) of them particularly delivered at Muhimbili National Hospital and the three Municipal hospitals respectively [31, 40]. The strength of this study is the fact that it involved multiple public hospitals, where the majority of deliveries occur, covering people with a diversity of demographic characteristics and health related behaviours. This study had limitations, one being the reliance on self-reported mother's information of the risk factors. The result is potential recall bias; however mother's whose newborns participated in the study either as a case or control were interviewed within 24 hours post-delivery to help minimize the bias. It was difficult for mothers to remember exactly at what gestational age they took which medication or they fell sick. While cigarette smoking and tobacco use have been widely implicated with birth defects, we could not examine these exposures in our study because none of our respondents reported cigarette or tobacco use [38, 41].

## Conclusion

This study identified maternal hypertension, maternal fever during pregnancy, and low birth weight newborns as risks factors associated with major structural birth defects in Dar es Salaam, Tanzania; while antimalarial use during pregnancy was associated with protection against major structural birth defects. By increasing early screening of pregnant mothers for hypertension and other causes of low birth weight or prematurity, through strengthening of the Primary Health Care system where antenatal care visits are done, it may be possible to reduce major structural birth defects in Dar es Salaam, Tanzania. In addition, we recommend promoting utilization of proven reproductive and child health and advocacy strategies to address the identified factors.
